# Aminopeptidases do not directly degrade tau protein

**DOI:** 10.1186/1750-1326-5-48

**Published:** 2010-11-05

**Authors:** K Martin Chow, Hanjun Guan, Louis B Hersh

**Affiliations:** 1 From the Department of Molecular and Cellular Biochemistry, University of Kentucky, Lexington, Kentucky, USA

## Abstract

**Background:**

Tau hyperphosphorylation and aggregation to form intracellular neurofibrillar tangles is prevalent in a number of tauopathies. Thus there is current interest in the mechanisms involved in Tau clearance. It was recently reported that Tau can be degraded by an aminopeptidase known as the puromycin sensitive aminopeptidase (PSA). Until now PSA has been reported to only cleave peptides, with the largest reported substrates having 30-50 amino acids. We have studied this unique PSA cleavage reaction using a number of different PSA preparations.

**Results:**

An N-terminally His tagged-PSA was expressed and purified from Sf9 insect cells. Although this PSA preparation cleaved Tau, product analysis with N and C terminal Tau antibodies coupled with mass spectrometry showed an endoproteolytic cleavage atypical for an aminopeptidase. Furthermore, the reaction was not blocked by the general aminopeptidase inhibitor bestatin or the specific PSA inhibitor puromycin. In order to test whether Tau hydrolysis might be caused by a protease contaminant the enzyme was expressed in E. coli as glutathione S-transferase and maltose binding protein fusion proteins or in Sf9 cells as a C-terminally His-tagged protein. After purification to near homogeneity none of these other recombinant forms of PSA cleaved Tau. Further, Tau-cleaving activity and aminopeptidase activities derived from the Sf9 cell expression system were separable by molecular sieve chromatography. When tested in a cellular context we again failed to see a PSA dependent cleavage of Tau. A commercial preparation of a related aminopeptidase, aminopeptidase N, also exhibited Tau cleaving activity, but this activity could also be separated from aminopeptidase activity.

**Conclusion:**

It is concluded that PSA does not directly cleave Tau.

## Introduction

The microtubule-associated protein tau (Tau) is located primarily in the central nervous system (CNS) and regulates the stability of microtubules. Tau is normally phosphorylated in cells with its state of phosphorylation related to developmental state [1]. Under abnormal conditions Tau becomes hyperphosphorylated, dissociates from microtubules and forms aggregates [2,3]. There are a number of neurodegenerative diseases caused by Tau aggregation collectively termed tauopathies. Among these is Alzheimer's disease in which intracellular Tau aggregates known as tangles are found in the brain and are believed to contribute to the etiology of the disease [4-6].

Like other microtubule-associated proteins (MAPs), a tandem microtubule-binding motif GSxxNxxHxPGGG is found at the C-terminus of Tau. Isoforms of Tau [7] have either 3 or 4 of these binding repeats due to alternative mRNA splicing of exon 10, which contains the fourth repeat. Additional variants of Tau are derived by an N-terminal insertion of exons 2 and 3, by insertion of only exon 3, and a form with no insertion. Together these variants result in six Tau isoforms in brain. The longest isoform (2N4R) contains exons 2 and 3 and 4 binding repeats (441 amino acids), while the shortest isoform (0N3R) has no N-terminal insert and 3 repeats (352 amino acids).

Due to its importance in neurodegenerative diseases, there have been a number of studies of Tau degradation by proteases such as the proteosome [8], caspase [9], and thrombin [10]. Recent reports suggest that the puromycin sensitive aminopeptidase (PSA, EC 3.4.11.14) may regulate Tau levels *in vivo *[11], and is able to hydrolyze Tau *in vitro *[12]. PSA has been characterized [13-15] as a zinc containing exopeptidase that sequentially cleaves the N-terminal amino acid from small peptides [16]. It is uniquely sensitive to micromolar concentrations of puromycin, hence its name. PSA is inhibited by the classical aminopeptidase inhibitor bestatin and its analogs. Until now PSA was thought to only cleave peptides containing no more than 30-50 amino acids. Thus Tau would be a novel substrate for PSA being considerably larger than any previously known substrate. The present study was designed to further investigate how PSA cleaves Tau; however, the results of these studies led us to conclude that Tau is not directly cleaved by PSA or by the closely related aminopeptidase, aminopeptidase N.

## Experimental Procedures

### Materials

PMSF, EDTA, bestatin, and *o*-phenanthroline were purchased from Sigma-Aldrich. Puromycin was from Invitrogen (Life Technologies). Monoclonal antibody (5A6) directed against N-terminal Tau residues 16 to 46 [17] was obtained from the Developmental Studies Hybridoma Bank developed under the auspices of the NICHD and maintained by The University of Iowa, Department of Biology. A C-terminal Tau antibody A0024, which is directed against the C-terminal ~1/3 of Tau, was obtained from DAKO.

### Cloning and Expression of Tau forms

Tau cDNAs in the pcDNA3.1 vector were generously provided by Dr. M. Hutton (Mayo Clinic, Jacksonville, Fl.). Two isoforms of Tau were used in this study: the longest isoform (Tau 2N4R) containing exons 2 and 3 as well as 4 microtubule binding repeats and Tau 0N4R containing 4 microtubule binding repeats without an N-terminal insert. For expression in E. coli, the 0N4R isoform-containing plasmid was amplified by PCR with the following primers:

Forward primer (5'-AATACATATGGCTGAGCCCCGC), which contains an Nde 1 site (underlined). Reverse primer (5'-GAATCTCGAGTTATCACAAACCCTGCTT), which removes the native stop codon and introduces an Xho 1 site (underlined) that fuses a C-terminal His_6 _tag.

PCR products were first subcloned into the pZero2 vector (Invitrogen) for sequencing, and then cloned into the pET32 vector at Nde1/Xho1 sites for expression.

To generate full-length Tau 2N4R in the pET32 bacterial expression vector, the sequence containing exons 2 and 3 was excised from pcDNA3.1-2N4R and inserted into pET32b-0N4R using BsiWI and HindIII restriction sites. These constructs were transformed in E. coli strain BL21*-*CodonPlus(DE3*)-*RP. For expression, cells were grown at 37°C until an OD_600 _of 0.5 to 0.7 was reached. Expression was then induced by the addition of isopropy-β-D-thiogalactoside (IPTG) to a final concentration of 0.5 mM and carried out for 3-4 hrs at 30°C. Cells were harvested and washed with PBS. Cell pellets were resuspended in 20 mM Tris-HCl, pH 7.5, containing 1 mM PMSF and 10 μM E-64 and broken with a French press. The crude homogenate was heated to 85°C for 8 min and centrifuged at 30,000*g *at 4°C for 30 min. The supernatant was then applied to a 5-ml His-Select Nickel affinity column (Sigma-Aldrich) and washed with the same buffer without protease inhibitors. Tau was eluted with 100 mM imidazole, concentrated, and the buffer exchanged to 20 mM Tris buffer pH 7.5, 1 mM DTT using a Millipore centricon. Both heat treated Tau [18] and His tagged Tau [12] have been shown to retain the ability to polymerize tubulin.

A stable tetracycline-inducible human Tau (2N4R) expressing cell line was obtained from Dr. Jeff Kuret (Ohio State University).

### Cloning and Expression of PSA

A full length human PSA cDNA [19] was subcloned into the pFastbac vector for expression as an N-terminal hexahistidine PSA (N-His_6_-PSA) in Sf9 insect cells [20]. To generate a C-terminal hexahistidine PSA (C-His_6_-PSA), a C-terminal fragment containing six His residues was amplified using PSA as a template with the following primers:

Forward (5'-TGCCCCCTGTGGATCGACTTGG-3'), preceding an internal Xho1 site, and reverse (5'-CGATAAGCTTTCAGTGGTGGTGGTGGTGGTGCACTGTGGGTGG-3'), containing a HindIII site (underlined).

This amplified 901 bp product and the 5' 1745 bp fragment were cloned into pFastbac1 using HindIII/Xho1 and Xho1/BamH1 sites, respectively. The procedures of transposing pFastbac-PSA to bacmid and subsequent baculovirus amplification were performed according to the manufacturer's instructions. Three days after infection with baculovirus, Sf9 cells containing recombinant enzymes were collected by centrifugation at 2,000*g *for 10 min., and frozen at -80°C until use. The procedure for purifying N-His_6_-PSA is similar to that as previously described [21]. Briefly, a cell paste was suspended in 10 volumes of lysis buffer (20 mM Tris buffer, pH 7.5, 1 mM PMSF and 10 μM E-64) and sonicated with a Branson Sonifier 450 (Output control at 4, Duty cycle 30%, 10 sec for 3 times). The homogenate was centrifuged 30 min at 30,000*g *and the supernatant applied to a Waters Acell Plus QMA anion exchange column (15 mL) in 20 mM Tris buffer, pH 7.5. Proteins were eluted with a 0 to 0.5 M linear NaCl gradient with PSA eluting at ~0.11 M NaCl. The eluted enzyme was concentrated and applied to a Pharmacia Superdex 200 column (1.6 cm × 60 cm) equilibrated and run with 20 mM Tris buffer, pH 7.5. The active PSA fractions were pooled, and concentrated with a Millipore Centricon concentrator and stored in 25% glycerol at -80°C.

For purifying C-His_6_-PSA, cell pellets were sonicated as described above with 300 mM NaCl and 5 mM imidazole included in the buffer. The supernatant was passed through a 5-ml His-Select Nickel affinity column and eluted with 100 mM imidazole. After concentration and buffer exchange the purified enzyme was made 20% glycerol and stored at -80°C.

PSA was also expressed in E. coli as GST and MBP fusion proteins. The expression vectors pET41, which contains a GST sequence and a TEV protease cleavage site and pETa-MBP [22], which contains a His_8_-Maltose binding protein and a TEV cleavage site were used. These plasmids were transformed into E. coli BL-21(DE3)-RP and the bacteria were allowed to grow until an OD_600 _of 0.5 to 0.7 was reached. PSA expression was induced by the addition of IPTG to a final concentration of 0.5 mM, and carried out overnight at 16°C, after which time cells were harvested, washed with PBS and stored at -20°C. A GST Prep FF 16/10 column (GE Health Science) was used to purify GST-PSA as previously described [21]. Purified GST-PSA was treated overnight with TEV protease at 4°C to remove GST and further purified by chromatography on an S-200 (GE Health Science) gel filtration column. GST-PSA, without being treated with TEV protease, was also chromatographed on the same column. His_8_-MBP-PSA was purified by chromatography on Ni resin as described above followed by S-200 chromatography. The active fractions were concentrated and treated with TEV protease to remove His_8_-MPB. Lastly the enzyme was purified on a 1-mL 15Q anionic exchange resin (GE Health Science).

### Lentivirus production

cDNAs for human PSA and an inactive E309A point mutant (PSAx) [20] were subcloned into the lentiviral vector pCSC-SP-PW. Lentiviruses were then produced using a four-plasmid transfection system as described previously [23].

### Aminopeptidase activity assays

Aminopeptidases were routinely assayed using 20 μM alanine 4-methoxy-2-naphthylamine (Ala-4-MNA) as substrate in 25 mM Tris-HCl buffer, pH 7.5. Free 4-MNA released by PSA was monitored on a fluorescent plate reader (GX, Molecular Device) at an excitation of 340 nm and an emission of 425 nm. Hydrolysis of Leu-enkephalin (YGGFL) was determined by reverse phase HPLC on a Phenomnex C_18 _column (5.0 × 4.6 mm) by following its disappearance at 214 nm. Products were separated by a linear gradient from 0.1% trifluoroacetic acid in 95% water/5% acetonitrile to 0.1% trifluoroacetic acid in 50% water/50% acetonitrile.

### Tau degradation in cells

HEK293 cells expressing a tetracycline-inducible human 2N4R Tau were grown in Dulbecco's modified Eagle's medium supplemented with 10% fetal bovine serum, 5 μg/ml blasticidin and 400 μg/ml zeocin [24]. For overexpression of human PSA, the cells were transduced with a PSA expressing lentivirus and cultured for 48 hrs. As controls, lentiviruses expressing GFP or an inactive point mutant of PSA (PSAx) were used. To induce Tau expression, 1 μg/ml tetracycline was added to the media and cells were collected 12 hrs later. Cells were lysed in 50 mM Tris HCl, pH 8.0, 150 mM NaCl, 1% NP40 containing complete protease inhibitors cocktail (Roche). The lysate was subjected to SDS-PAGE and Western blot analysis.

### Electrophoresis and Western Blot Analysis

SDS-PAGE [25] was conducted using a Bio-Rad Protean system. Proteins were stained with Coomassie Blue R250 (Sigma-Aldrich). The BioRad Precision Plus All Blue Protein standard was used for estimating molecular weight. Density of protein bands were assessed using ImageJ software [26]. For Western analysis, gels were transferred onto a polyvinyldifluoride membrane (Millipore) according to Towbin [27]. The dilutions of primary antibodies were 1:5,000 for antibody 5A6 and 1:8.000 for antibody A2400. Proteins were detected and quantified using a Li-COR Odyssey Imaging system with the following secondary antibodies: Alexa Fluor 680 goat anti-mouse IgG (Life Technologies) and IRDye 800 conjugated affinity purified goat anti-rabbit IgG (Rockland).

### Protein assay

Protein concentration was determined using Coomassie Plus (Pierce Chemical Company). Bovine serum albumin was used as the protein standard.

### Identification of protein fragments by Mass Spectrometry

Protein bands were identified by mass spectrometry at the University of Kentucky Proteomics core within the Center of Structure Biology. This facility is supported in part by grant P20RR020171 from the NIH NCRR. Briefly, gel bands were excised from SDS-PAGE, digested with Sigma proteomics grade trypsin, and alkylated with iodoacetamide. Samples were then analyzed with an Applied Biosystems 4800 MALDI TOF/TOF Proteomics Analyzer. MALDI MS spectra were used for analyzing Tau products. For unknown proteins, MS/MS spectra were used to search against the Uniprot_sprot_20100125+ contams.fasta database through Protein Pilot (Applied Biosystems) Ver. 2.0, Rev. 50861.

## Results

**Figure 1:**
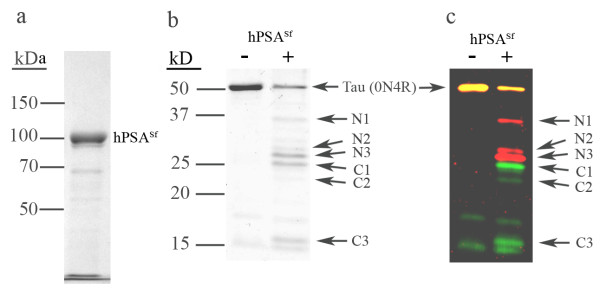
**Human PSA^sf ^preparation cleaves Tau**. (a) Purity of hPSA^sf ^(5 μg). (b) Degradation of Tau by hPSA^sf^. Tau was incubated with PSA at a molar ratio of 15:1 (Tau:PSA) in 10 mM Tris buffer, pH 7.5, 1 mM DTT at 37°C for 16 h. Reactions containing 1 μg Tau were analyzed by SDS-PAGE on a 12.5% gel. Labeled products and intact Tau were further analyzed by mass spectrometry and the result is shown in Table 1. (c) Reaction products immunoblotted with N-terminal Tau antibody 5A6 (red) or C-terminal Tau antibody A00024 (green). Twenty percent of the reaction mix was loaded in each lane. Intact Tau shows a yellow color due to the red/green overlap. N1 also reacts to A00024, but was overshadowed by higher intensity signal of 5A6. Molecular weight markers in kDa are shown at the left.

We initially tested the reaction of purified recombinant human PSA expressed in Sf9 insect cells with Tau. This form of PSA was expressed as a fusion protein containing an N-terminal hexahistidine affinity tag (N-His_6_-hPSA) as previously described [20]. The purity of N-His_6_-hPSA was estimated to be ~90% as judged from a Coomassie stained SDS-PAGE gel, figure 1a. After removal of the affinity tag this purified hPSA (hPSA^Sf^) was tested for its ability to hydrolyze Tau by incubation with recombinant Tau 0N4R for 16 hours at 37°C at a molar ratio of Tau to PSA of 15:1 as described by Karsten *et al*. [11]. Cleavage of Tau was apparent as assessed by Coomassie blue protein staining of the reaction subjected to SDS-PAGE, figure 1b. To further analyze the reaction, major products shown in figure 1b were analyzed by peptide mass spectral fingerprinting. The identified peptides from these products are listed in Table 1. Additionally, degradation products were reacted with antibodies specific to the N-and C-terminal regions of Tau (figure 1c). Since hPSA^Sf ^is an exopeptidase that cleaves amino acids sequentially from the N-terminus, we were surprised to find that reaction products, N1 to N3, which were decreased in size by apparent molecular weights of greater than 15 kDa, still retained the epitopes for the N-terminally directed antibody 5A6. The identified peptides (Table 1) agree with that of Western analysis in that the N-terminus of these cleavage products is intact. This finding shows that Tau cleavage was internal, not expected for an aminopeptidase.

**Table 1:** Identification of Tau degradation products by mass spectral peptide fingerprinting.

Protein Band	Calc'd (M+H^+^)	Expt'l (M+H^+^)	Peptides identified
N1	2069.89	2069.67	^6^QEFEVMEDHAGTYGLGDR
	2181.90	2181.74	^25^DQGGYTMHQDQEGDTDAGLK
	2424.13	2424.89	^45^AEEAGIGDTPSLEDEAAGHVTQAR
	1393.63	1393.50	^137^SGYSSPGSPGTPGSR
	842.55	842.43	^217^VQIINKK
	1980.09	1979.87	^241^HVPGGGSVQIVYKPVDLSK

N3	2069.89	2069.81	^6^QEFEVMEDHAGTYGLGDR
	2424.13	2424.05	^45^AEEAGIGDTPSLEDEAAGHVTQAR
	1393.63	1393.61	^137^SGYSSPGSPGTPGSR
	1066.59	1066.55	^154^TPSLPTPPTR
	842.55	842.50	^217^VQIINKK

C1	1420.78	1420.64	^154^TPSLPTPPTREPK
	842.55	842.48	^217^VQIINKK
	1974.00	1973.90	^264^CGSLGNIHHKPGGGQVEVK
	1578.82	1578.76	^296^IGSLDNITHVPGGGNK
	978.54	978.51	^318^LTFRENAK
	1101.55	1101.52	^338^SPVVSGDTSPR
	1381.66	1381.61	^381^QGLLEHHHHHH

C3	842.55	842.44	^217^VQIINKK
	1578.82	1578.67	^296^IGSLDNITHVPGGGNK
	978.54	978.46	^318^LTFRENAK
	1101.55	1101.45	^338^SPVVSGDTSPR
	1381.66	1381.54	^381^QGLLEHHHHHH

**Figure 2:**
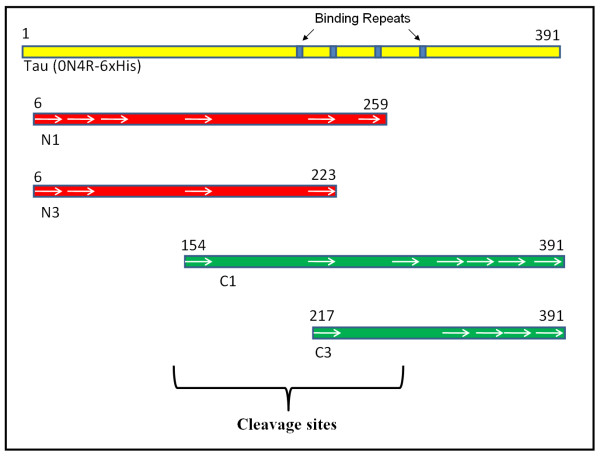
**Diagrammatic presentation of Tau (0N4R) degradation and the possible cleavage regions**. Degradation products were drawn based on Western analysis (Figure 1b). Identified peptides (Table 1) from the corresponding protein bands (Figure 1a) are shown as white arrows.

We also identified four C-terminal peptides derived from the PSA cleavage reaction (C1 through C4). Although these fragments could result from sequential amino acid release from the N-terminus, the number and size of the bands detected by the C-terminal Tau antibody A0024 makes this unlikely. This follows since one would expect a series or ladder of products detected by the C-terminal antibody rather than discreet peptides. figure 2 schematically superimposes the identified peptides on the Western analysis and shows that the cleavage sites are located in the vicinity of the four microtubule-binding motif repeats.

**Figure 3:**
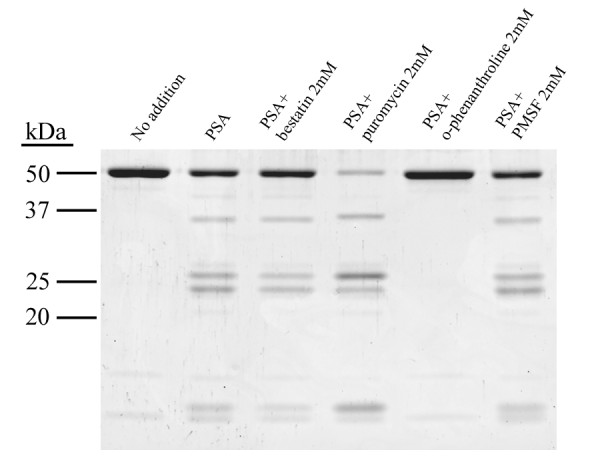
**Hydrolysis of Tau by hPSA^Sf ^in the presence of protease inhibitors**. hPSA^sf ^was preincubated with protease inhibitors on ice for 15 min, and then tested for activity towards Tau as described in Figure 1.

We found that the hPSA^Sf ^dependent cleavage of Tau was not blocked by the aminopeptidase inhibitors bestatin and puromycin, but was inhibited by the metal chelator *o*-phenanthroline (figure 3). Puromycin rather than inhibit had an activation effect on the reaction. As a control we showed that the reaction of hPSA^Sf ^with two known PSA substrates, alanine 4-methoxy-2-naphthylamide (Ala-4-MNA) and Leu-enkephalin, was completely inhibited by bestatin and puromycin under the same conditions in which these inhibitors failed to inhibit Tau cleavage, (Table 2). The finding of endopeptidase-like cleavage of Tau, the insensitivity of the reaction to PSA inhibitors, together with the sensitivity of the reaction to the metal chelator *o*-phenanthroline suggest either contamination of the hPSA^Sf ^preparation with a metallo-endopeptidase that cleaves Tau or that the cleavage of Tau by hPSA^Sf ^proceeds through a novel mechanism.

**Table 2:** Inhibition of PSA^sf ^Tau cleaving activity by protease inhibitors

Substrate	% Activity Remaining			
	**Bestatin**	**Puromycin**	***o*-phenanthroline**	**PMSF**
	**(2 mM)**	**(2 mM)**	**(2 mM)**	**(2 mM)**

Alanine 4MNA	0	0	0	79

Dynorphin A	0	0	0	79

PSA^sf ^was preincubated with protease inhibitors at 2 mM on ice for 15 min. PSA activity was then assayed with alanine 4MNA, Dynorphin A. PSA^sf ^incubated with buffer was taken as 100% to determine the remaining PSA activity of each treatment.

**Figure 4:**
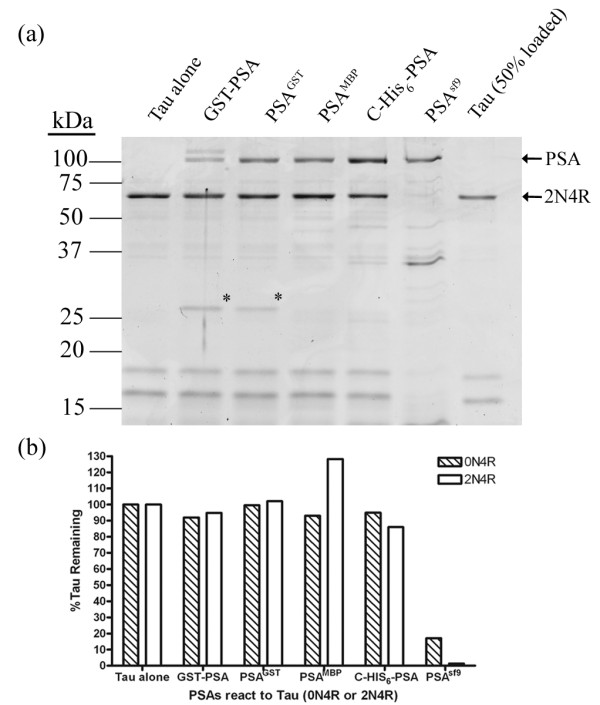
**Reaction of Tau with various forms of PSA**. Same amount of PSA activity was reacted with Tau 2N4R or Tau 0N4R in 10 mM Tris buffer, pH 7.5, 1 mM DTT at 37°C for 16 h. (a). Reaction mixtures were analyzed by SDS-PAGE, only Tau 2N4R is shown. (b). For quantitation, the intensity of Tau remaining was determined using ImageJ software [26]. For internal control, a 50% Tau alone (last lane) was run and the band intensity shown to be ~50% of the input Tau (first lane). Further, by analyzing the staining intensity of the PSA bands we calculated that the variation in the amount of PSA added in each lane varied less than 4%. For the GST-PSA lane, both GST-PSA and free PSA were present. Their intensities were combined to get total PSA. * = free GST.

In an attempt to resolve this issue we expressed hPSA in *E. coli *assuming that if the cleavage of Tau was due to a contaminating protein derived from Sf9 cells it would be highly unlikely that the same or similar contaminant would be present in enzyme preparations derived from a totally different system. We thus expressed hPSA as two different fusion proteins, a glutathione S-transferase fusion protein (GST-hPSA) and a-maltose binding protein fusion protein (MBP-hPSA). Since N-His_6_-hPSA did not bind well to a nickel resin and was purified conventionally, an additional form of PSA containing a C-terminal His_6 _tag (C-His_6_-PSA) was expressed in Sf9 insect cells and purified on a nickel resin. The specific activities of all of the PSA preparations derived from Sf9 cells or *E. coli *were essentially the same, ~6 μmol of 4-MNA released from Ala-4-MNA per min per mg protein. Equal amounts of PSA activity from these different preparations was tested for their ability to cleave full-length Tau 2N4R, since it has been previously reported [11,12] that the 2N4R isoform is cleaved by hPSA^GST ^with or without the GST removed. We used the same reaction conditions as described [11], which included 10 mM Tris buffer, pH 7.5 and 1 mM DTT. When tested, with the exception of the original hPSA^Sf ^(N-His_6_-hPSA with His tag removed), none of these other PSA preparations cleaved Tau 2N4R, figure 4. An identical result was obtained when Tau 0N4R was used as a substrate, figure 4b. Thus, it is very likely that the N-His_6_-hPSA preparation, being the only one of four different PSA preparations that cleaved Tau, contains a contaminating protease responsible for Tau cleavage.

**Figure 5:**
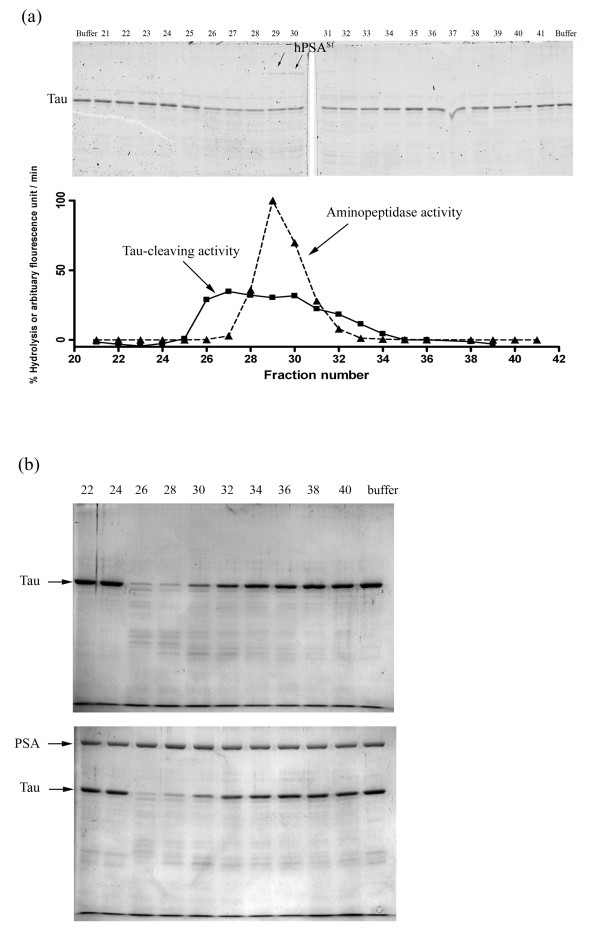
**Separation of aminopeptidase and Tau-cleaving activities on an S-200 column**. (a). PSA derived from Sf9 cells was purified as described in Methods. Following an anionic QMA column, the enzyme was chromatographed on an S-200 column and fractions were assayed for both Tau and aminopeptidase activity. The arrows show the eluted PSA activity detected on the gel. Tau activity is shown as the % hydrolysis (solid square), while aminopeptidase activity toward alanine 4-MNA is shown by solid triangles. (b). An Sf9 cell extract from non infected cells was fractionated the same way as the above. Upper panel: Fractions assayed for Tau-cleaving activity. Lower panel: Fractions assayed for Tau-cleaving activity in the presence of exogenous C-His_6_-PSA with a molar ratio of Tau:PSA (5:1).

Next, we attempted to resolve PSA activity from Tau cleaving activity in the N-His_6_-hPSA^Sf ^preparation. A preparation of N-His_6_-hPSA^Sf ^was purified from an Sf9 cell extract following the purification scheme described in Methods. At the final molecular sieve purification step PSA activity toward the synthetic substrate Ala-4-MNA and Tau cleaving activity were assayed in fractions collected from the S200 column. As shown in figure 5a, Tau activity was eluted in a broad peak with maximal activity at fraction 27. In contrast true PSA activity as measured with Ala-4-MNA peaked at fraction 29. Despite the fact that elution of Tau cleaving activity exhibits a broad peak that overlaps with PSA activity, these activities are clearly distinguishable. For example, Tau cleaving activity is approximately the same in fractions 27 and 29, but aminopeptidase activity in fraction 29 was ~35 times higher than that of fraction 27.

As a further control we examined Tau cleaving activity in extracts from untransfected Sf9 cells taken through the same purification scheme. As shown in figure 5b (upper panel) Tau cleaving activity was detected in a similar elution profile in naive cells as seen when PSA was expressed. This shows the presence of endogenous Tau cleaving activity in Sf9 cells that likely contaminated our purified N-His_6_-hPSA^Sf ^preparation. To determine whether PSA, rather than cleave Tau, might activate Tau cleavage by a contaminating protease, we added C-His_6_-PSA, known to have aminopeptidase activity but no Tau cleaving activity, to each of the Tau cleaving untransfected Sf9 derived fractions. As seen in figure 5b (lower panel), the Tau degradation profile was not altered by the additional of C-His_6_-PSA.

We attempted to identify other protein bands in the hPSA^Sf ^preparation that might cleave Tau by subjecting the N-His_6_-hPSA^Sf ^preparation to SDS-PAGE, cutting out gel slices, and using peptide mass spectral fingerprinting to identify protein contaminants. Although there were trace proteins present, due to the lack of a *Spodoptera frugiperda *protein database, only an insect heat shock protein (~70 kd) was identified as a contaminant based on its homology to the *Cercopia *moth protein.

**Figure 6:**
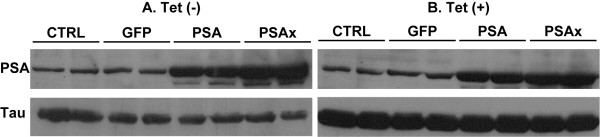
**PSA does not cleave Tau in cells**. HEK293 cells expressing a tetracycline-inducible 2N4R Tau were transduced with lentivirus expressing GFP, PSA or its inactive mutant form (PSA_x_) and cultured for 48 hrs. Tetracycline (1 μg/ml) [Tet(+)] or vehicle (1:1 vol/vol water/ethanol) [Tet(-)] was added and cells were collected 12 hrs later. Cell extracts were subjected to SDS-PAGE, and analyzed by Western blot analysis with goat anti-hPSA (Chemicon) at 1:2000, and mouse anti-Tau antibody 5A6, at 1:4000. This was followed by incubation with peroxidase conjugated secondary antibodies and ECL Western Blotting detection.

To determine whether PSA might facilitate cleavage of Tau in a cellular context, we transduced HEK 293 cells expressing a tetracycline-inducible human 2N4R Tau with a lentivirus expressing PSA. As controls lentiviruses expressing GFP or an inactive point mutant of PSA (PSAx) were used. The expressed PSA had no effect of the intracellular level of Tau under steady-state expression conditions (figure 6A) or when Tau expression was induced (figure 6B).

**Figure 7:**
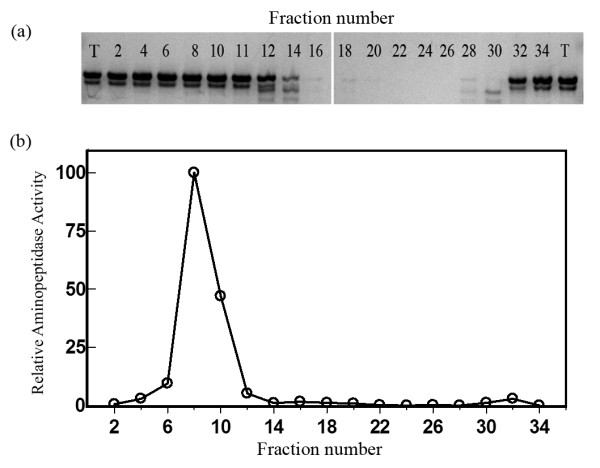
**Separation of aminopeptidase N from Tau-cleaving activity**. A commercial preparation of aminopeptidase N was fractionated on a butyl S-650 hydrophobic chromatography. (a). Tau-cleaving activity was estimated by the intensity of the remaining Tau band as analyzed by SDS-PAGE. T = Tau with buffer only. (b) Aminopeptidase N activity as determined with alanine 4MNA as substrate.

We tested the reactivity of another aminopeptidase, aminopeptidase N (ApN) with Tau because it is a related protein with very similar kinetics and substrate specificity to PSA [16]. Surprisingly, we found that a commercial purified porcine ApN preparation cleaved Tau. To determine whether the Tau cleaving activity is caused by ApN, we fractionated this ApN preparation by hydrophobic chromatography on a TosoHaas butyl-650 column and tested the reactivity of the eluted fractions toward Tau and toward the aminopeptidase substrate Ala-4-MNA. The results showed that aminopeptidase activity and Tau-cleaving activity are clearly resolved (figure 7), demonstrating that aminopeptidase N does not hydrolyze Tau. Attempts to identify the Tau-cleaving enzyme by mass spectral fingerprinting also met the same difficulty as with the insect protein, namely a lack of an adequate database. Unfortunately only ~300 porcine DNA sequences are currently deposited in the NCBI database, and none of those showed up in our analysis. Thus the identification of the Tau cleaving enzymes derived both Sf9 cells and from porcine kidney remains unknown, but is clearly not PSA.

## Discussion

In this study several lines of evidence have been obtained which show that the puromycin sensitive aminopeptidase does not directly cleave Tau. (i) The cleavage pattern obtained with Sf9 derived N-His_6_-hPSA is inconsistent with aminopeptidase cleavage. (ii) Several PSA preparations derived using different expression systems and purification methods do not cleave Tau. Only the preparation obtained from Sf9 cells containing an N-His_6_-affinity tag cleaves Tau. The N-His_6 _affinity tag containing PSA was the only form that was not purified by affinity chromatography. (iii) Aminopeptidase and Tau-cleaving activity can be separated by gel filtration chromatography for PSA and by hydrophobic chromatography for the related aminopeptidase N. (iv) Within a cellular context increasing PSA expression did not affect Tau steady-state levels. Thus, we are forced to conclude that PSA does not cleave Tau and that the Tau-cleaving activity we observed in our PSA preparation is caused by an as yet unidentified contaminating protease. Since this activity was inhibited by *o*-phenanthroline this contaminating protease is likely a metalloprotease.

Our finding that PSA does not cleave Tau appears at variance with several published reports [11,12,28]. Although our purified PSA preparations, including the same GST fusion protein previously studied [11] did not cleave Tau, it is still possible that PSA regulates Tau cleavage by an indirect mechanism. Studies in TauP301L transgenic mice in conjunction with a functional analysis in *Drosophila *associate Tau as being regulated by PSA [11] and a recent report showed that using an interference RNA against PSA increased Tau levels in SH-SY5Y cells [28]. However as shown in this study over expression of PSA in HEK cells did not change Tau levels. Since we conclude that PSA does not directly cleave Tau, the regulation of Tau levels by PSA might work through its interaction with other proteins or by regulating the level of a peptide that facilitates Tau cleavage. For example it has been shown that PSA works in conjunction with the proteosome dependent degradation of polyglutamine containing proteins by cleaving the released polyglutamine peptides [29]. Understanding the mechanism by which PSA affects Tau levels will clearly require additional studies.

## Abbreviations

PSA: Puromycin-sensitive aminopeptidase; GST: Glutathione S-transferase; MBP: maltose binding protein; E-64: trans-Epoxysuccinyl-L-leucylamido(4-guanidino)butane; DDT: dithiothreitol; MNA: 4-methoxy-2-naphthylamide; PMSF: phenylmethylsulfonylfluride

## Competing interests

The authors declare that they have no competing interests.

## Authors' contributions

HG and KMC performed all the experiments. KMC and LBH participated in the experimental design, data analysis and manuscript drafting. All authors read and approved the manuscript.
